# A Path From Sustainable Nutrition to Nutritional Sustainability of Complex Food Systems

**DOI:** 10.3389/fnut.2019.00039

**Published:** 2019-04-12

**Authors:** Sergiy M. Smetana, Sabine Bornkessel, Volker Heinz

**Affiliations:** ^1^German Institute of Food Technologies (DIL e.V.), Quakenbrück, Germany; ^2^Faculty of Agricultural Sciences and Landscape Architecture, University of Applied Sciences, Osnabrück, Germany

**Keywords:** nutritional sustainability, artificial intelligence, LCA, food ecodesign, personalized diet, complex food systems

## Abstract

Integration of nutritional and sustainable aspects is a complex task tackled by a few scientific concepts. They include multiple dimensions and functions of food systems trying to provide solutions for harmonic co-evolution of humanity and planet Earth. “Nutritional Sustainability” is differentiated from other concepts which combine nutrition and sustainability as it not only sets environmental sustaining capacity as a baseline level for balanced nutrition, but also aims for the search of food system driving nodes. It does not aim for the support of solutions of producing enough or more food for increasing population (sustainable nutrition), neither does it contradict other similar concepts [sustainable nutrition security, nutritional life cycle assessment (LCA)]. However, it calls for more definite estimation of the carrying capacity of the environment on personal, local, and national levels for the development of more efficient solutions of nutrition balanced in the limits of environmental carrying capacity. The review is providing a few examples of advances in nutritional science (personalized nutrition, nutrigenetics), food technology (personalized food processing, food ecodesign), and food complex systems (artificial intelligence and gut microbiome), which have a great potential to progress sustainable food systems with Nutritional Sustainability set as a guiding concept.

## Introduction: Feeding the World Population Sustainably

Food systems, defined as compositions of interlinked elements and activities aimed for the production, processing, distribution, and consumption of food (1), have been considered to include sustainability aspects for a long time (2). The appearance of sustainability concepts (3) rather quickly and seamlessly transferred to the nutrition research (4) forming the field of Sustainable Nutrition Security (5, 6). During the last few decades an increasing number of studies and industrial applications address the topics of supplying more proteins, carbohydrates and energy (4) to feed the increasing population mainly by means of intensive agriculture. Sustainable nutrition, therefore, included research on topics supplying enough resources to grow feed and food for current state and future generations (7); searching efficient solutions to deal with malnutrition (producing more from less) (8) and finding ways for more balanced nutrition and dealing with obesity (producing less from more) (9).

Nutritional science developed a sophisticated knowledge base on balanced diets (10), which are aimed to promote healthier state of human organisms. “Sustainable diets” approach on the other hand from the initial appearance additionally included avoidance of excessive use and degradation of natural resources (11). Further development of “sustainable diet” approach resulted in inclusion of three main components (social, economic, and environmental) and eventually bigger number of elements each representing a complex system (12, 13). Increased complexity in nutritional and sustainability research and need to account for multiple aspects led to the development of two main approaches: First is indicator-based accounting for the key parameters for the development of a universal sustainable diet (1) and second is accounting for the hidden interdependencies with a complex system analysis (14, 15).

At the same time, behavioral attitude of people is not always following the recommendation for healthy or sustainable diets (16). Recent studies on the personalization of nutrition based on phenotypic or genotypic information demonstrate the lack of evidence on behavioral changes toward the recommendations (17–19). Similarly, a lack of evidence exists for the demonstration of behavioral change of consumers toward less environmentally impacting foods. Sustainability of food products is at the bottom of the list of important criteria when buying foods in supermarkets (20). Therefore, the lack of evidence on the effect of dietary and sustainability recommendations indicate that there is a need for new leverages for the enhancement of social behavior and societal transition to healthier and more sustainable food consumption.

**Figure 1:**
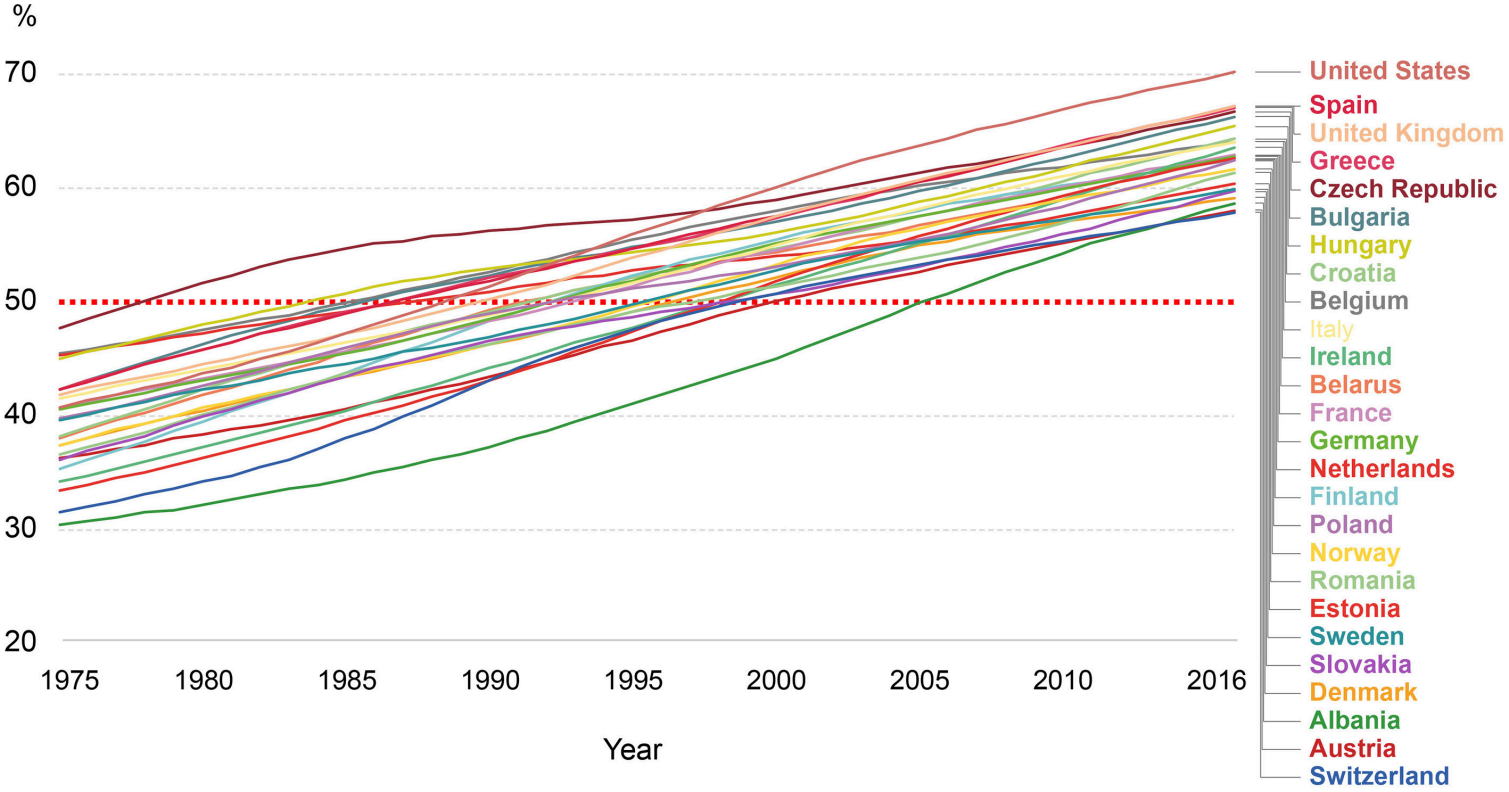
Share of adult population in some Western countries (18 years and more) who have a body-mass index (BMI) ≥25 (adapted from ourworldindata.org, based on data from WHO, Global Health Observatory).

Absence of efficient means to change the behavior of consumers in developed countries toward healthier and environmentally friendly diets implies the persistence of high rates of food consumption in the future. Statistical data indicate that in Western countries there are higher consumption rates per capita of food rich in simple carbohydrates and fats (21). It leads to the overwhelmed rates of obesity and overweight (Figure 1), which cannot be related to the overall educational or awareness levels of society, but rather to education inequalities within countries (22, 23). The overconsumption behavior of Western populations might be a consequence of potentially misleading approach of nutrition security (24) or “Sustainable Nutrition,” when the need to supply more foods at lower price results in nutritional shift toward processed foods and convenience products.

It became obvious that existing ways to deal with more sustainable ways to supply adequate amounts of food to growing population cannot be fulfilled in the limits of resources of planet Earth. “Business as usual” approach in nutrition leads to consumption of resources beyond safe planetary boundaries (25). Regional diversity of socio-economic and resource availability conditions further complicates “safe operating boundaries” (26, 27). High-income countries have high impacts on the environment associated with high food waste generation at consumer level and with health-impacting nutrient consumption (28). Low-income countries, on the other hand, have higher impacting problems associated with harvesting food losses, food availability and affordability, safety and socio-cultural well-being (29). Indicated problems call for system solutions, when not only separate technological or societal issues are changed, but also a considerable improvement in the whole system is achieved. Food systems, therefore, are perceived as social–ecological systems, formed of biophysical and social factors linked through feedback mechanisms comprising the activities involved in food production, processing, packaging, distribution, retail, and consumption at different scales (social, economic, political, institutional and environmental processes, and dimensions) (15, 30, 31). Only considering the multiple elements and connections at different scales it is possible to account for indirect and rebound effects in system solutions. Currently, there are a few conceptual approaches suggested as a basis for the system change: socio-technical regimes and sustainability transitions (32, 33), resilience of systems and sustainability transformations (34), “Sustainable Nutrition Security” as multiscale multi-dimension policy-oriented methodology (6, 35). At the same time, “sustainable diet” or “healthy diet” approaches set as a guiding principle for sustainable food systems are not fulfilling the criteria of complex system transformation (36), and therefore are not calling for efficient transformation of the entire food system, but rather for niche solutions.

In this paper, the main studies exploring connections between “nutrition” and “environmental impact” with different approaches will be overviewed. We performed a literature search in open literature databases and search engines of “Google Scholar,” “Mendeley” and “WorldWideScience” in 2018, beginning 2019 using the terms “nutrition,” “environmental impact,” “Life Cycle Assessment,” “food system,” “complex system,” “complex system control,” “sustainable nutrition,” “sustainable diet.” The search aimed for original studies, case studies, reviews, or highlights pointing at the connection between nutrition, human health and environment and emerging solutions allowing the transformation of complex food systems. The references of the articles found were also explored for consistency. The findings highlighting the potential for transformation of complex food systems will be discussed.

## Healthy Population and the Environment

Emergence of Life Cycle Assessment (LCA) in food field supported the statements of finding ways for more efficient production to feed future growing population. However, recent studies concentrate more and more on the need to combine indirect food impact (environmental impact with LCA) with direct impact of food (allergies, toxins, nutrition). They concentrate on the need to reveal the direct food impact (nutritional properties) as a form of environmental influence. Nutritional studies, at the same time, start concentrating on issues of balanced diet for healthy population and “low” nutrition diets (sugar, carbohydrates, fat, or calories) for groups with special dietary requirements (overweight, obese). This way, two separate concepts originating from LCA of food and nutrition: (1) dealing with direct and indirect environmental impacts; and (2) (re-)balanced nutritional diets are leading to the change of perception from “Sustainable Nutrition” to “Nutritional Sustainability.” A very useful definition of Nutritional Sustainability is offered by Swanson et al. (37): “Nutritional sustainability is the ability of a food system to provide sufficient energy and the amounts of essential nutrients required to maintain good health of the population without compromising the ability of future generations to meet their nutritional needs.” It is interesting that innovations related to nutritional sustainability are often associated with product development in the pet food industry, which is more inclined to use alternative biomass sources and by-products from “human food system” (37–40).

Most of the studies, dealing with sustainability and nutrition, even though point out to that need to approach the food system holistically (1, 13, 14, 29, 41–43), rarely relate to the findings of complex system analysis and complex system control theories (36, 44), thus suggesting niche solutions, not able to transform the entire system. That is why, we considered further development of “Nutritional Sustainability” highlighting potential paths for food system change. Nutritional sustainability is an ability of human communities [as key driving nodes (36)] to find ways of complex food system transformation toward limited consumption of natural resources within regional or planetary boundaries while fulfilling own nutritional needs. The definition includes a few main components: human communities (groups, populations) as elements with highest degree of distribution (36), boundaries outlining the transformation aim of complex food system in current state and in the future and multiscale approach (from regional to global). Nutritional sustainability, therefore, allows to concentrate on defining specific nodes on each level, capable of guiding the dynamics of the whole complex system.

Emergence of “Nutritional Sustainability” is seamlessly changing the future scope of nutritional science, LCA of food and potentially resulting in formation of a new joined concept field. There are a few signs, which indicate the formation of a new area of sustainability research. Several recent studies aiming for the assessment of nutrition and diets rely on more complex nutritional basis (comparing to previously used mass, energy, or protein content-based units). Researchers emphasize on the need to account the consumption of right amount of food for the establishment of healthy diets (42, 43, 45). Others put efforts for the development of methodologies for the combination of environmental and direct food impacts on health (41, 46) or target wide scope of sustainable indicators related to food production and consumption (5, 29). Such trends identify the need for the combination of knowledge on joined environmental and direct social effects with efficient communication and nudging approaches to force the behavioral change.

A fair question from the reader would be on importance of conceptual change from “Sustainable Nutrition” and “Sustainable Nutrition Security” (6, 12) to “Nutritional Sustainability.” What changes might the emergence of “Nutritional Sustainability” bring in the future for the research, industries, and society? We believe that such a conceptual shift in thinking is associated with the need to define specific action nodes with high degree of influence (driver nodes) capable of system transformation to a new state with defined boundaries. Currently, the most adequate system boundaries of safe human operations are outlined with the concept of planetary boundaries (26). More radical concepts of sustainability are currently required, as existing “soft” and “gradual” approaches are not able to coop with rapidly evolving environmental problems (47, 48). For example, research activities aiming for more radical solutions could be “blocked” by the legal and ethical established practices. Even though, the General Data Protection Regulation (49), which got in force in 2018, is aimed to protect human rights for safety of personal information, it creates multiple obstacles for the clinical and nutrition related research activities in EU. Even though it does not directly block the use of bibliometric, health and genetic related data for research purposes, it creates multiple challenges and obstacles in working with such data (50–52). Moreover, raised ethical issues on the influence of digital marketing create ethical limitations on the use of data from social and digital networks (53). Legal obstacles also exist in terms of nutritional human research related to novel food sources such as insects and microalgae (54–57). These are only a few of the limiting examples for the acceptance of Nutritional Sustainability as a guiding concept for the research and practice in food systems.

## Shifting Complex Food System to the Desired State

The concept of Nutritional Sustainability can also be beneficial for recent research activities in multiple fields. Personalization of nutrition as a potential driving node of a complex system is already foreseen as one the most important topics to solve problems associated with elderly nutrition (aging population in Western countries) and special dietary needs (allergies, intolerances, overweight, and malnutrition), which is reflected in increasing number of studies (58). Moreover, personalized adapted food production (e.g., reformulation) or “personalized food processing” (e.g., additive manufacture) could result in more resource efficient food production with reduced amount of food wasted (59). Determination of specific dietary requirements based on nutrigenomic and nutrigenetic data (60, 61) and current state of an organism in combination with precise fulfillment of such needs could potentially lead to more balanced and healthy nutrition with minimized environmental impact.

Another emerging approach connected with the concept of Nutritional Sustainability is transferring from industrial ecology. Ecodesign of food can be a viable conceptual methodology, when used not only from the perspective of using less resources to produce food, but also in terms of designing potential food reuse or recycle. It is well-recognized that LCA is more effective as a tool if applied at the stage of design. Despite some rare examples (62), ecodesign is not applied in food industry, due to the absence of data on the future production and consumption processes of a developed product. Moreover, food products are developed with safety, nutritional properties, shelf stability in mind, but almost never with potential for recycling or upcycling. The design phase is currently not accounting for the need to separate packaging, components of meals, and disintegrate biomass for further use. Ecodesign of food (not only food technologies) has a potential of transformation of food waste management. Therefore, food waste treatment is becoming a complex technologically intensive problem, which could be solved at the beginning of the chain with integration of ecodesign principles in food product development (as a key driving node capable of shifting complex system). Integration of ecodesign principles in the upstream stages of food production could lead to more efficient food waste treatment.

Studies, associated with the topic of nutrition and sustainability, more often indicate the upcoming influence of artificial intelligence methods (63, 64). The developments are not limited to the identification of properties, but also oriented toward the analysis of dynamic health effects, often acquired with multiple personal health tracking devices appearing on the market. Emergence of nutrigenomics and identification of connections between genetic preconditions and food impact on health through machine learning techniques (61) is giving a new boost for the development of nutritional sustainability. Such approach can result in supplying defined amount of personally tailored food and in lowering of environmental impact (resource and waste reduction).

Furthermore, direct impact of food on health is correlated with human gut microbiome. It is well-known that microbiome is in a great degree defining the health of a human organism. Healthy functioning of gut microbiome from one side depends on the diet and from the other supports proper functioning of metabolism and nutrition, physiology and immune system. Unbalanced gut microbiome can cause severe gastrointestinal conditions such as inflammatory bowel disease and irritable bowel syndrome, and further force development of obesity, type 2 diabetes, and atopy (65). Recent studies indicate that pre-biotics might not be as suitable for the reinforcement of microbiome in all the cases as previously believed (66). It is also well-known that food and diets affect different people in diverse ways. Multiple other studies indicate that gut microbiome might be structured and function differently under different conditions, which call for studies on complex system analysis, e.g., multiomics and time series measurements (67). A great diversity of functions, compositions of gut microbes and human personalized attributes create a necessity to find a viable approach applicable for the analysis of their interference as a complex system (68). Machine learning and artificial intelligence algorithms can analyze complex systems of gut microbiome and human health personalized responses in real time and provide applicable recommendations (63). Complex multilayer interaction between food consumed, gut microbiome, health effects, and current advances achieved with application of artificial intelligence indicate the emergence of another driving node for transition of food systems.

The complexity of food-human-sustainability interaction is connected not only to multiple components, but also to the rapid expansion of the system. Human health is a category of direct impact of nutrients and environment, but not limited to those components. Social components of health care, life style, culture and traditions (to name a few) as complex systems themselves substantially complicate the food system. Then the estimation of safe operating boundaries of different scales requires not only smart artificial intelligence algorithms, but also a proper modeling of complex systems (36, 64), dynamic multilayer networks (69), and ecodesign principles.

## Outlook

Nutritional Sustainability is differentiated from other concepts combining nutrition and sustainability via setting environmental sustaining capacity as aiming point, achievable via changing of key driving nodes for transformation of food systems. Current advances in nutritional science, food technologies, and complex food systems indicate that such a concept is emerging in the nearest future. Nutritional Sustainability concept does not contradict to other similar concepts (sustainable nutrition security, nutritional LCA), but it calls for more definite estimation of the carrying capacity of the environment on personal, local, and national levels and identification of key driving nodes able guiding the dynamics of entire system. Without such system boundaries and key driving nodes, theoretical and practical solutions would be limited in efficiency.

## Author Contributions

SS, SB, VH contributed equally to conception and design of the study. SS and SB performed literature review. SS wrote the first draft of the manuscript. SB wrote sections on nutrition of the manuscript. VH provided input in sustainable food system design. All authors contributed to manuscript revision, read, and approved the submitted version.

### Conflict of Interest Statement

The authors declare that the research was conducted in the absence of any commercial or financial relationships that could be construed as a potential conflict of interest.
